# Pointing Hand Stimuli Induce Spatial Compatibility Effects and Effector Priming

**DOI:** 10.3389/fpsyg.2013.00219

**Published:** 2013-04-26

**Authors:** Akio Nishimura, Chikashi Michimata

**Affiliations:** ^1^Department of Psychology, Laboratory of Cognitive Psychology, Sophia UniversityTokyo, Japan; ^2^Japan Society for the Promotion of ScienceTokyo, Japan

**Keywords:** spatial compatibility, perception and action, inter-individual interaction, hierarchical coding, effector priming, pointing

## Abstract

The present study investigated the automatic influence of perceiving a picture that indicates other’s action on one’s own task performance in terms of spatial compatibility and effector priming. Participants pressed left and right buttons with their left and right hands respectively, depending on the color of a central dot target. Preceding the target, a left or right hand stimulus (pointing either to the left or right with the index or little finger) was presented. In Experiment 1, with brief presentation of the pointing hand, a spatial compatibility effect was observed: responses were faster when the direction of the pointed finger and the response position were spatially congruent than when incongruent. The spatial compatibility effect was larger for the pointing index finger stimulus compared to the pointing little finger stimulus. Experiment 2 employed longer duration of the pointing hand stimuli. In addition to the spatial compatibility effect for the pointing index finger, the effector priming effect was observed: responses were faster when the anatomical left/right identity of the pointing and response hands matched than when the pointing and response hands differed in left/right identity. The results indicate that with sufficient processing time, both spatial/symbolic and anatomical features of a static body part implying another’s action simultaneously influence different aspects of the perceiver’s own action. Hierarchical coding, according to which an anatomical code is used only when a spatial code is unavailable, may not be applicable if stimuli as well as responses contain anatomical features.

## Introduction

Other’s actions affect one’s own cognitive processing and task performance. For example, the perceived direction of another’s eye gaze is widely known to elicit reflexive attentional shifts, even when the gaze direction is non-predictive or counter-predictive (Friesen and Kingstone, 1998; Driver et al., 1999; Ristic and Kingstone, 2005; Galfano et al., 2012). Eye gaze has also been shown to activate responses on the side of its direction, eliciting the spatial compatibility effect (Ansorge, 2003; Zorzi et al., 2003; Ricciardelli et al., 2007): the phenomenon in which performance is better when a spatial stimulus feature (such as a location or symbolic spatial meaning) is congruent with a spatial response feature than when they are incongruent, irrespective of whether the spatial stimulus feature is relevant to the task (Kornblum et al., 1990; Simon, 1990; Umiltà and Nicoletti, 1990; Lu and Proctor, 1995; Hommel and Prinz, 1997; Proctor and Vu, 2006). Additionally, humans tend to imitate the gaze direction of other individuals (Ricciardelli et al., 2002).

In daily life, referential pointing with an extended index finger is ubiquitous. The index finger pointing gesture is used as a social cue to communicate spatial information; the performer’s intent to indicate spatial direction and/or location might be stronger than his or her eye gaze (Burton et al., 2009). Recently, Ariga and Watanabe (2009) reported reflexive attentional shifts elicited by pointing pictures. Participants localized a target that appeared to the left or right. Before the presentation of the target, a leftward or rightward hand stimulus, whose direction was non-informative, was briefly presented. A larger attentional cueing effect was observed for hand stimuli with the index finger extended than for hand stimuli with the little finger extended, with the index and middle fingers extended, or with no finger extended (i.e., a fist) during short stimulus onset asynchronies (SOAs; 107 ms). In addition, the attentional cueing effect was smaller for hand stimuli with the index finger shortened to the length of the little finger, or with the little finger lengthened to the length of the index finger, than for the normal index finger pointing stimuli. These findings suggest that directional body parts affect a viewer’s attention, and that the index finger pointing gesture is selectively strong during this process.

Perceiving a pointing hand stimulus would affect not only attention but also action. The spatial compatibility effect is one such case. Studies have found that the spatial compatibility effect (Eimer, 1995; Ansorge, 2003; Zorzi et al., 2003; Wühr and Kunde, 2006; Ricciardelli et al., 2007; Nishimura and Yokosawa, 2010a) can be induced by lateral and central stimuli that induce automatic attentional shifts, such as the sudden appearance (Posner, 1980) or disappearance (Theeuwes, 1991) of stimuli, eye gaze (Friesen and Kingstone, 1998; Driver et al., 1999; Ristic and Kingstone, 2005; Galfano et al., 2012), and arrows (Hommel et al., 2001b). Likewise, the pointing hand stimuli are expected to prime actions on the side of pointing direction and to elicit the spatial compatibility effect.

Another possible influence of the perception of a pointing hand stimulus on action is effector priming: perceiving another’s body parts could prime an observer’s action using the same body part. Recent studies have shown that left/right anatomical identity of the presented hand stimulus affects responses using the left or right hand (Ottoboni et al., 2005; Vainio and Mustonen, 2011).

Thus, the pointing hand stimulus could potentially affect manual responses in two ways: the spatial compatibility effect by its *spatial* meaning and effector priming by its *anatomical* hand identity. However, whether these two effects could emerge simultaneously is unclear. Concerning the horizontal (i.e., left/right) response coding in the spatial compatibility effect with spatial stimulus features, Heister et al. (1990) proposed the hierarchical coding hypothesis of the horizontal spatial response. According to the hierarchical coding hypothesis, the spatial code of the response location (left button vs. right button) is ranked higher than, and is used in priority to, the anatomical code of the effector identity (left hand vs. right hand) to represent the response as left or right. The lower-ranked internal coding of anatomical identity is influential only when a higher-ranked external positional coding could not be used. Evidence for hierarchical action coding is reported in a wide range of interactions between spatial/spatially-associated stimulus features and manual responses (Klapp et al., 1979; Müller and Schwarz, 2007; Nishimura and Yokosawa, 2010b).

However, there might not be a hierarchical relationship between spatial and anatomical coding themselves, if stimulus as well as action properties are considered. Hierarchical coding might not be applicable when a stimulus also has anatomical features. Automatic imitation – a tendency to perform the same movement using the body part corresponding to the perceived body movement – emerges simultaneously with the spatial compatibility effect (Bertenthal et al., 2006; Catmur and Heyes, 2011). This suggests that both the spatial and the effector-based effects could be simultaneously observed in an appropriate situation. Action coding along multiple dimensions (Hedge and Marsh, 1975; Nicoletti and Umiltà, 1984; Rubichi et al., 2006), based on multiple action effects (Hommel, 1993, 1996; Grosjean and Mordkoff, 2002), and based on both vertical spatial and horizontal anatomical features (Nishimura and Yokosawa, 2010b) also supports the potential availability of multiple levels for action coding.

The present study investigated the influence of perceiving a pointing hand stimulus on one’s own manual response action in terms of spatial compatibility based on symbolic/spatial features and effector priming based on anatomical features. We used a task similar to the Simon task (Simon, 1990; Lu and Proctor, 1995) in which the compatibility-related and effector-related stimulus features were task-irrelevant, in order to test automatic influences (see also Ottoboni et al., 2005). Participants were required to make left or right button press responses based on the color of a centrally presented target patch while ignoring a task-irrelevant hand picture. The hand pictures displayed a left or right hand with the index or little finger extended. The direction of the extended finger was either left or right. Participants pressed the left and right button with their left and right hands, respectively. Spatial compatibility was based on the relationship between the pointing direction and the response location (left vs. right): compatible when the pointing direction and the response location corresponded and incompatible when they were opposite. On the basis of Ariga and Watanabe’s (2009) findings regarding attentional shift, we predicted that both the index- and little-finger pointing stimuli should elicit the spatial compatibility effect, and that the compatibility effect should be larger for the pointing index finger than for the pointing little finger.

Effector priming was based on the relationship between the anatomical identity (left hand vs. right hand) of the pointing hand and the response hand. If symbolic/spatial feature coding based on the environmental reference frame is ranked higher than, and is used in priority to, anatomical feature coding in the cognitive processing hierarchy (Heister et al., 1990), then pointing direction alone should affect performance: the spatial compatibility effect should be present but the effector priming effect should be absent. In contrast, if the symbolic/spatial and anatomical properties could simultaneously influence cognitive processing, then both pointing direction and hand identity should affect performance: both the spatial compatibility effect and the effector priming effect should be observed. To further investigate whether effector priming is modulated by postural congruency between the presented body part and the body part used for responding, we asked participants to press the response buttons by using their extended index fingers for one block and their extended little fingers for another block, while the other fingers were folded. The participants’ hand posture was congruent with the observed hand posture if the index (little) finger pointing was displayed while the participants used their index (little) fingers for responding. The participants’ hand posture was incongruent with the observed hand posture if the index (little) finger pointing was displayed while the participants used their little (index) fingers for responding.

## Experiment 1

In Experiment 1, we examined the automatic influence of briefly presented task-irrelevant hand stimuli depicting leftward or rightward pointing with the little or index finger on a manual horizontal button-pressing task, from the perspective of spatial compatibility between the pointing direction and the location of the response button and effector priming between the pointing hand and the identity of the response hand. The pointing hand picture was briefly presented 160 ms prior to the presentation of the target. We used a short SOA because attentional cueing effects were observed with an SOA of 107 ms but not with an SOA of 1,000 ms in the study by Ariga and Watanabe (2009).

### Method

#### Participants

Twenty-four volunteers (20 females; mean age = 24.1 years; all right-handed) participated in this experiment. All participants reported normal or corrected-to-normal vision.

#### Apparatus and stimuli

Experiments were controlled by MATLAB 7.2 (MathWorks). Visual stimuli were presented on a 24′′ LCD display (Diamondcrysta RDT241WEX, Mitsubishi). The left and right shift keys were used as response keys. Participants pressed the left and right response keys with their left and right index fingers, respectively, in one block and with their left and right little fingers in another block. The experiment was conducted in a darkened room.

**Figure 1:**
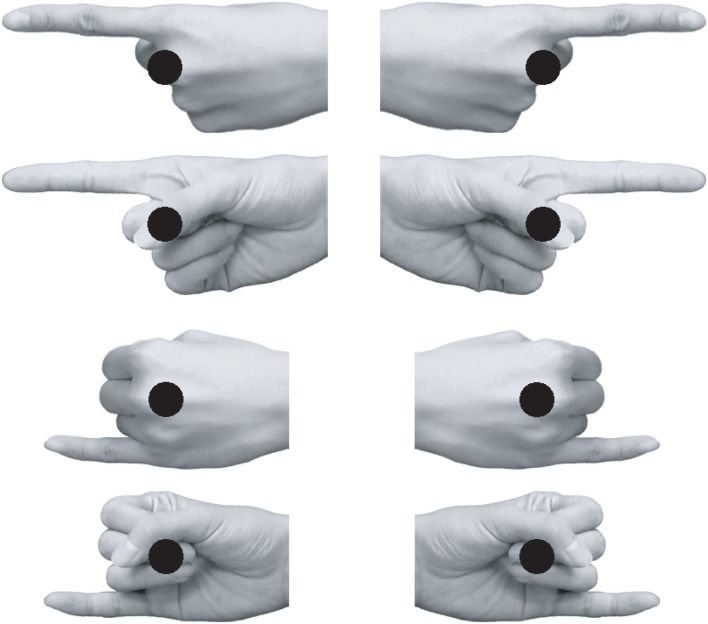
**Examples of pointing hand stimuli**. The black dot superimposed on each hand represents the position of the target relative to the hand. Note that the target was superimposed on the hand stimulus only in Experiment 2. In Experiment 1, the hand stimulus disappeared before the onset of the target dot.

Visual stimuli were presented at the center of the display on a gray background. The fixation point consisted of a white dot (3 mm in diameter). Target stimuli were a green or red dot (11 mm in diameter). The pointing hand stimuli (92–101 mm width × 39–51 mm height for the index finger pointing; 75–76 mm width × 40–50 mm height for the little finger pointing) were grayscale palm or back hand images with the index or little finger extended while the other fingers were clenched (Figure 1). Hand stimuli were obtained from three females and three males. Eight types of finger pointing stimuli, a combination of view (back or palm), pointing finger (index or little finger), and pointing direction (left or right; mirror-reversed images were used), were used for each model. Thus, 48 images in total were used as pointing hand stimuli. A chin rest maintained a viewing distance of 60 cm.

#### Task and procedure

Participants were instructed to press the left or right response key based on the color of the target as quickly and accurately as possible. Half of the participants were required to press the left key for green targets and the right key for red targets. The other half was required to press the left key for red targets and the right key for green targets.

Each trial started with the presentation of the white fixation point. The duration of the fixation point ranged from 1,000 to 2,000 ms. Then, a pointing hand was presented for 60 ms. After an inter-stimulus interval (ISI) of 100 ms, the central target dot was presented until the response was made. The inter-trial interval (ITI) preceding the next trial was 1,000 ms. During the ISI and the ITI, the gray background was presented on the screen.

The experiment had two experimental blocks. Each block contained 288 trials of 3 replications for each combination of pointing hand view (2; back, palm), pointing finger (2; index finger, little finger), pointing direction (2; left, right), model of the pointing hand (6), and target stimulus color (2; green, red). Trial order was randomized. Participants were given a short break every 48 trials, after which they restarted the experiment with a left or right shift key press. They used their left and right index fingers to press the left and right response keys in one block and their little fingers in another block. Response finger order was counter-balanced across participants. A practice block of 16 trials preceded each experimental block.

### Results

**Figure 2:**
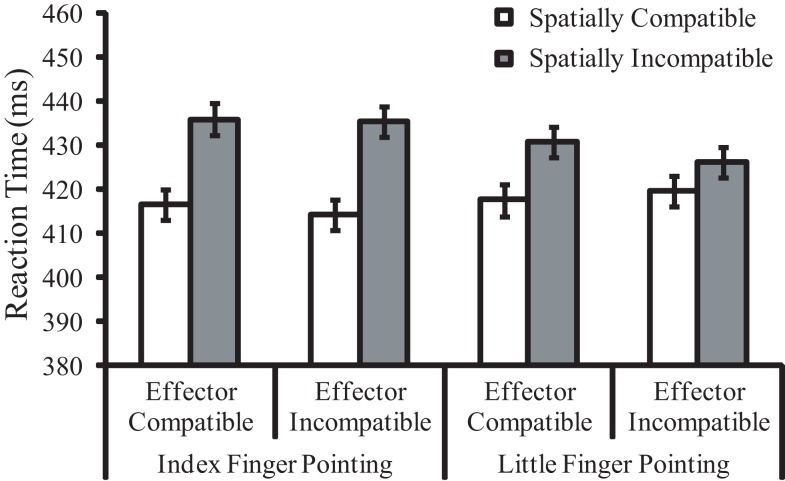
**Mean reaction times (in milliseconds) for Experiment 1 as a function of pointing finger, effector compatibility, and spatial compatibility**. Error bars represent within-subjects standard errors pooled from the three factors.

Trials in which reaction times (RTs) were less than 100 ms or more than 1,000 ms (<1% of all trials) were excluded as outliers from the RT analyses. The overall error rate was low (2.5%) and therefore immaterial to our discussion. The error rate pattern was generally consistent with RT results (Table 1 and Figure 2). Mean RTs for correct responses were submitted to an analysis of variance (ANOVA) with pointing finger of the hand stimulus (index finger, little finger), spatial compatibility between the left/right pointing direction and the left/right response key position (compatible, incompatible), effector compatibility between the left/right identity of the pointing hand stimulus and the left/right identity of the response hand (compatible, incompatible), and postural (i.e., extended finger) congruency between the pointing hand stimulus and the responding hand (congruent, incongruent) as within-subjects factors.

**Table 1:** **Mean reaction time (ms) and error rate (%; in parentheses) for Experiments 1 and 2 as a function of pointing finger, spatial compatibility, effector compatibility, and postural congruency**.

Pointing finger		Index		Little	
Spatial compatibility		Compatible	Incompatible	Compatible	Incompatible
Experiment 1				
Postural congruency	Effector compatibility				
Congruent	Compatible	412 (1.0)	428 (3.6)	423 (2.2)	436 (2.7)
	Incompatible	409 (2.0)	430 (2.8)	427 (2.2)	432 (4.5)
Incongruent	Compatible	421 (1.4)	443 (3.4)	412 (1.5)	426 (3.0)
	Incompatible	420 (1.3)	440 (3.6)	412 (1.6)	420 (3.7)
Experiment 2				
Congruent	Compatible	402 (1.7)	410 (2.5)	398 (1.7)	396 (0.9)
	Incompatible	406 (1.7)	418 (1.6)	401 (1.6)	404 (2.5)
Incongruent	Compatible	393 (1.2)	408 (2.4)	404 (1.4)	403 (1.9)
	Incompatible	401 (0.9)	409 (4.7)	407 (1.5)	412 (3.0)

The main effect of spatial compatibility was significant, *F*(1, 23) = 25.89, *p* < 0.001, $\eta_{p}^{2}=0.53$, indicating a 15 ms spatial compatibility effect. Responses were faster when the pointing direction and the response key position were congruent (*M* = 417 ms) than when they were incongruent (*M* = 432 ms). The two-way interaction between spatial compatibility and pointing finger of the hand stimulus was significant, *F*(1, 23) = 9.62, *p* = 0.005, $\eta_{p}^{2}=0.29$. The spatial compatibility effect was larger for the pointing index finger (20 ms, *p* < 0.001) than for the little finger (10 ms, *p* = 0.002). The two-way interaction between pointing finger and postural congruency was significant, *F*(1, 23) = 7.12, *p* = 0.014, $\eta_{p}^{2}=0.24$, reflecting faster index finger key presses (*M* = 419 ms) than little finger key presses (*M* = 430 ms). Other main effects or interactions, including the main effect of effector compatibility (−1 ms effector priming effect), were not significant (*p*s > 0.05).

### Discussion

We found evidence of the spatial compatibility effect. Performance was better when the pointing direction of the task-irrelevant pointing hand stimulus was consistent with the response location than when the pointing direction was opposite to the response location. The spatial compatibility effect was larger for the pointing index finger than for the little finger. These findings are consistent with the previous evidence demonstrating superiority of the pointing index finger as an attention-directing pointing gesture (Ariga and Watanabe, 2009).

In contrast to the spatial compatibility effect, the effector priming effect was not observed in Experiment 1. This might indicate that when the spatial compatibility effect due to symbolic/spatial feature overlap emerges, no effector-based effect due to anatomical feature overlap emerges. However, recent studies have shown that time is needed for the effects related to body parts to develop (Catmur and Heyes, 2011; Vainio and Mustonen, 2011). Therefore, the brief presentation of the pointing hand stimulus might be responsible for the absence of the effector priming effect in Experiment 1. To test this issue, we extended the duration of the pointing hand stimulus in Experiment 2.

## Experiment 2

In Experiment 2, to maximize the possibility of observing the effector priming effect (see Vainio and Mustonen, 2011), the SOA between the pointing hand stimulus and the target dot was extended to 560 ms (from 160 ms in Experiment 1). Additionally, duration of the pointing hand stimulus was extended until a response was made. Therefore, the pointing hand stimulus remained present even after onset of the target dot. If symbolic/spatial feature coding based on the environmental reference frame is ranked higher than, and used in priority to, anatomical feature coding in the cognitive processing hierarchy (Heister et al., 1990), only the spatial compatibility effect should be observed. However, if the symbolic/spatial and anatomical properties can simultaneously influence cognitive processing, but effector priming needs some time to occur (Vainio and Mustonen, 2011), then both the spatial compatibility effect and the effector priming effect should be observed in this experiment.

### Method

#### Participants

Twenty-four undergraduate and graduate students (14 females; mean age = 22.4 years; 22 right-handed) participated in this experiment. All participants reported normal or corrected-to-normal vision. None of them had participated in Experiment 1.

#### Apparatus, stimuli, task, and procedure

Stimuli and procedures were the same as Experiment 1 except for the following: the pointing hand stimuli were presented until a response to the red or green dot was made. The target stimulus was superimposed upon the pointing hand stimulus (see Figure 1). The SOA between the pointing hand stimulus and the target was 560 ms.

### Results

**Figure 3:**
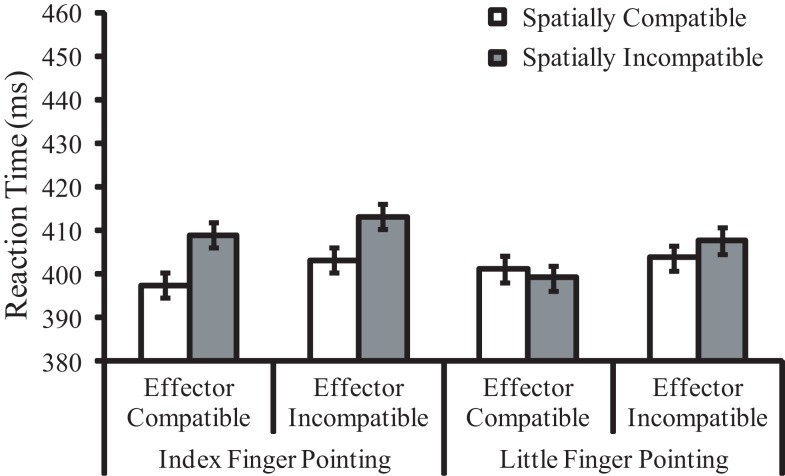
**Mean reaction times (in milliseconds) for Experiment 2 as a function of pointing finger, effector compatibility, and spatial compatibility**. Error bars represent within-subjects standard errors pooled from the three factors.

Outliers (<1% of all trials; defined using the same criteria as in Experiment 1) were excluded from the analyses. As in Experiment 1, the overall error rate was low (2.0%) and therefore immaterial to our discussion. The error rate pattern was generally consistent with RT results (Table 1 and Figure 3). Mean RTs for correct responses were submitted to an ANOVA with the pointing finger (index finger, little finger), spatial compatibility (compatible, incompatible), effector compatibility (compatible, incompatible), and postural congruency (congruent, incongruent) as within-subjects factors.

The main effect of spatial compatibility was significant, *F*(1, 23) = 12.61, *p* = 0.002, $\eta_{p}^{2}=0.35$, indicating a 6 ms spatial compatibility effect. Responses were faster when the pointing direction and the response key position were congruent (*M* = 401 ms) than when they were incongruent (*M* = 407 ms). The main effect of effector compatibility was also significant, *F*(1, 23) = 19.95, *p* < 0.001, $\eta_{p}^{2}=0.46$, indicating a 5 ms effector priming effect. Responses were faster when the pointing hand and the response hand had the same left/right identity (*M* = 402 ms) than when the identity was reversed (*M* = 407 ms). The two-way interaction between spatial compatibility and pointing finger was significant, *F*(1, 23) = 14.79, *p* < 0.001, $\eta_{p}^{2}=0.39$. The spatial compatibility effect was larger for the pointing index finger (11 ms, *p* < 0.001) than for the little finger (1 ms, n.s.). Other main effects or interactions were not significant (*p*s > 0.05).

### Discussion

With longer presentation of the task-irrelevant pointing hand stimuli than in Experiment 1, both the spatial compatibility and the effector priming effects emerged in Experiment 2. As in Experiment 1, the spatial compatibility effect between the pointing direction of the hand and the response location was observed. However, the spatial compatibility effect was significant only for the pointing index finger. In contrast to Experiment 1, the effector priming effect was observed in Experiment 2. Performance was better when the task-irrelevant pointing hand was anatomically identical to the hand used for the button press response (e.g., left hand pointing and a key press with the left hand) than when they were different (e.g., left hand pointing and a key press with the right hand). The effector priming effect was not modulated by postural congruency between the pointing hand and the responding hand.

Experiment 2 revealed that with sufficient duration, both pointing direction and anatomical identity of a task-irrelevant pointing hand stimulus could simultaneously affect an observer’s action. This finding is consistent with the notion that the absence of effector priming effect in Experiment 1 was due to insufficient time to develop rather than hierarchical coding of symbolic/spatial and anatomical features.

## General Discussion

The present study investigated the automatic influence of symbolic/spatial and anatomical features of task-irrelevant pointing hand stimuli on manual key press responses in terms of spatial compatibility between pointing direction and response location and of effector compatibility between the pointing hand and the response hand. Although only the spatial compatibility effect was observed in Experiment 1 (brief presentation of the pointing hand stimuli prior to target onset), both the spatial compatibility and the effector priming effects emerged in Experiment 2 (longer SOA and duration of the pointing hand stimuli). Thus, we revealed that with sufficient processing time, pointing hand pictures could automatically affect observer’s actions based on both symbolic/spatial and anatomical features. An imitative compatibility effect between viewing and doing *dynamic* manual actions emerges simultaneously with the spatial compatibility effect (Bertenthal et al., 2006; Catmur and Heyes, 2011). The present study showed the simultaneous occurrence of spatial compatibility effect and effector priming effect based on left/right anatomical identity even when stimuli (pointing hand pictures) and responses (manual button presses) were both *static*.

Hierarchical horizontal action coding, in which spatial coding is superior to anatomical coding, has been repeatedly confirmed in interactions between spatial/spatially-associated stimulus features and horizontal manual actions (Klapp et al., 1979; Heister et al., 1990; Müller and Schwarz, 2007; Nishimura and Yokosawa, 2010b). However, the simultaneous occurrence of the spatial compatibility and effector priming effects suggests that hierarchical coding is not applicable, and that action coding in terms of both spatial (location) and anatomical (effector identity) features can be simultaneously influential, when the stimulus also has anatomical feature. We conclude that there is no hierarchical relationship between spatial coding and anatomical coding themselves. Both the spatial and effector-based effects could simultaneously emerge in appropriate situations (see also Bertenthal et al., 2006; Nishimura and Yokosawa, 2010b; Catmur and Heyes, 2011). The present findings suggest the importance of considering stimulus properties in addition to action properties to understand the action coding in interaction between perception and action.

No interactions involving both spatial compatibility and effector compatibility were significant in the analyses. The spatial compatibility effect was larger in Experiment 1 than in Experiment 2, whereas the effector priming effect was observed only in Experiment 2. Furthermore, the size of the spatial compatibility effect did not correlate with the size of the effector priming effect (*r* = 0.00 for Experiment 1 and *r* = 0.02 for Experiment 2). Although inconclusive, these results suggest that the spatial compatibility and effector priming effects emerge independently.

Although the spatial compatibility and effector priming effects may emerge with independent processing, these effects are commonly explained in terms of ideomotor theory. According to this theory (James, 1890; Prinz, 1997; Hommel et al., 2001a), actions are represented and controlled by their perceptual consequences (action effects). This notion implies that perception/cognition and action control use common representation. Therefore, the stimulus features should activate the actions with corresponding features through the action effect codes. Manual button presses accompany perceptual events within the visual, auditory, proprioceptive, and tactile senses at the response location with the effector used for that response (see Hoffmann et al., 2009). In the present study, spatial information conveyed by the pointing direction of the hand stimulus should activate the corresponding spatial code of left or right, also associated with action on that side. As a result, a response was facilitated when the pointing direction was on the side of the correct response, but response conflict emerged when the pointing direction was opposite the correct response because the pointing direction activated the code representing the incorrect response. Similarly, a stimulus depicting left (or right) hand should activate representation of that hand, which is also used to control action with the hand. This activation facilitated the actions using the left (or right) hand, inducing the effector priming effect.

### Spatial compatibility effect

The spatial compatibility effect was observed in the present study. Performance was better when the direction of the pointing hand stimulus and the response location were on the same side than when they were on opposite sides. In Experiment 1, with a brief presentation of the pointing hand stimulus and a short SOA of 160 ms, the spatial compatibility effects for both the index and the little finger pointing hand stimuli were significant, but the former was larger than the latter. Ariga and Watanabe (2009) revealed larger reflexive shifts of attention for the pointing index finger than for the pointing little finger following a brief presentation of a pointing hand stimulus with SOA of 107 ms. Thus our spatial compatibility findings add further evidence that stimuli eliciting reflexive shifts of attention also elicit the spatial compatibility effect (see the Introduction for other examples), and are consistent with the notion of a close relationship between attention and action (e.g., Rizzolatti et al., 1987; Rubichi et al., 1997; Deubel et al., 1998; Humphreys and Riddoch, 2005).

In Experiment 1, the spatial compatibility effect was larger for the pointing index finger than for the pointing little finger. In Experiment 2, the spatial compatibility effect was significant for the pointing index finger but not for the pointing little finger. The superiority of the index finger in the spatial compatibility effect was constant across both experiments (10 ms). One possible cause of the larger spatial compatibility effect in index finger pointing is intentionality. Previous studies suggest that in the interaction between individuals, the effect of the perceived action of another on one’s own task performance should be larger when the action is recognized as intentional (Tsai and Brass, 2007; Liepelt et al., 2008; Liepelt and Brass, 2010; Atmaca et al., 2011). The index finger pointing hands may be special stimuli from which the spatial intention/meaning of the actor is automatically extracted, probably because an index finger pointing gesture is widely used to indicate spatial position or direction, whereas a hand with a little finger extended is rarely used for such purposes.

Another possible cause of the difference in spatial compatibility effects between the index and little finger pointing is morphological differences between the index and little finger pointing stimuli such as finger length, extended position, or size. Our study alone cannot distinguish these two possibilities, but Ariga and Watanabe (2009) shed some light on this issue. Although their paradigm differed from the present one, they obtained cueing effects of similar magnitude for hand stimuli with the little finger extended, with the index and middle fingers extended, with the index finger shortened to the length of the little finger extended, or with the little finger lengthened to the length of the index finger extended. These effects were smaller than that of the normal index finger pointing stimuli. This finding suggests that the larger spatial effect related to the index finger pointing hand stimuli is specific to normal index finger pointing and cannot be explained by position or length of the extended finger or by the overall size of the picture. Taken together, we tentatively conclude that the spatial compatibility effect specific to the pointing index finger should be based on the spatial intention/meaning automatically extracted from a picture of a body part that implies an action. However, a further experiment would be required to rule out the possibility that similar effects would be seen with non-social stimuli of similar shape and size.

### Effector priming

An effector priming effect between the left/right anatomical identity of the presented task-irrelevant pointing hand and that of the hand used for the button press was absent with a 160-ms SOA and brief presentation of pointing hand stimuli (Experiment 1). However, effector priming effect was present with a 560-ms SOA and longer presentation of hand stimuli until response (Experiment 2). Thus, the present study showed that the effector priming effect required additional time to develop. This finding suggests that it takes a certain amount of time for the anatomical left/right feature of the perceived hand to be identified, and/or that it takes time for that feature to affect manual action using the left/right hand. In addition, absence of the influence of postural congruency indicates that feature codes that represent effector identity and control manual movements are posture-free, at least in the range of those used in the present study.

Vainio and Mustonen (2011) reported the effector priming effect with manual button press responses according to a target superimposed on centrally presented task-irrelevant hand stimulus. Their results were similar to those obtained in the present study. In their study, the effector priming effect was present with SOAs of 400 ms and 700 ms, but was not reliable with 100 ms SOA. Most hand postures of the stimuli used in their studies elicited similar effector priming effects. However, the direction of the hand modulated effector priming: positive effector priming effect emerged for the upward hand (wrist on the bottom), whereas negative effector priming effect was found for the downward hand (wrist on the top). We obtained a positive effector priming effect with in-between hand direction (i.e., leftward/rightward hand). Identification of boundary condition(s) of positive/negative effector priming in future research will support further understanding of the automatic influence of perceiving body parts on viewer’s actions in various ways and with various functions, such as the integration of perceived information and motor processes for action control, action mirroring, and communication (e.g., Liepelt et al., 2010; Vainio and Mustonen, 2011).

The present study obtained evidence for effector priming based on anatomical identity with static stimuli and responses. Properties of the effector priming effect, such as the requirement of adequate time to emerge (see also Vainio and Mustonen, 2011) and simultaneous occurrence with spatial compatibility effects, were consistent with those of imitative compatibility effects based on the correspondence of body parts and their movements (Bertenthal et al., 2006; Catmur and Heyes, 2011). Further research is needed to determine the elements specific to movement in the interaction between the perception of body parts and use of corresponding body parts.

## Conclusion

The present study investigated the influence on one’s own task performance of perceiving body parts that imply another’s action using a presentation of task-irrelevant pointing hand stimuli and manual button press responses. A spatial compatibility effect between the pointing direction and response location and an effector priming effect between the left/right anatomical identity of the pointing hand and response hand simultaneously emerged. For example, when a right hand with an index finger pointed to the left was presented, response on the left side and response using the right hand were activated. Our findings on spatial compatibility effects and on effector priming were consistent with the literature on each topic. We conclude that even when spatial directional information is delivered by a stimulus implying another’s action, anatomical features of the action are also automatically extracted, and that both the spatial/symbolic and the anatomical features simultaneously influence different aspects of one’s own action. The hierarchical coding account (Heister et al., 1990), according to which anatomical features are used only if spatial features are unavailable, was not supported when stimuli as well as responses involved corresponding anatomical features.

## Conflict of Interest Statement

The authors declare that the research was conducted in the absence of any commercial or financial relationships that could be construed as a potential conflict of interest.
